# PGC-1-Related Coactivator Modulates Mitochondrial-Nuclear Crosstalk through Endogenous Nitric Oxide in a Cellular Model of Oncocytic Thyroid Tumours

**DOI:** 10.1371/journal.pone.0007964

**Published:** 2009-11-23

**Authors:** Mahatsangy Raharijaona, Soazig Le Pennec, Julie Poirier, Delphine Mirebeau-Prunier, Clothilde Rouxel, Caroline Jacques, Jean-Fred Fontaine, Yves Malthiery, Rémi Houlgatte, Frédérique Savagner

**Affiliations:** 1 INSERM, UMR 915, l'institut du Thorax, Nantes, France; 2 Université de Nantes, Nantes, France; 3 INSERM, UMR 694, Angers, France; 4 Université d'Angers, Angers, France; 5 CHU Angers, Laboratoire de Biochimie, Angers, France; Victor Chang Cardiac Research Institute (VCCRI), Australia

## Abstract

**Background:**

The PGC-1 related coactivator (PRC), which shares structural and functional features with PGC-1α, is believed to regulate several metabolic pathways as well as mitochondrial biogenesis. Its involvement in the early programming of cell proliferation suggests the existence of finely regulated crosstalk between mitochondrial functions and the cell cycle status.

**Methodology/Principal Findings:**

PRC-regulated pathways were explored in a cell-line model derived from mitochondrial-rich tumours with an essentially oxidative metabolism and specifically high PRC expression. The functional status of mitochondria was compared to the results of microarray analysis under conditions of temporal PRC inhibition. To specify the fine PRC regulation, the expression levels of the genes and proteins involved in the oxidative phosphorylation process were studied by real time quantitative PCR and western blotting. As in earlier studies on PGC-1α, we investigated the role of nitric oxide in PRC-regulated mitochondrial biogenesis and determined its action in the control of the phosphorylation status of the mitogen-activated protein kinase pathway.

**Conclusion/Significance:**

We found that nitric oxide rapidly influences PRC expression at the transcriptional level. Focusing on mitochondrial energetic metabolism, we observed that PRC differentially controls respiratory chain complexes and coupling efficiency in a time-dependent manner to maintain mitochondrial homeostasis. Our results highlight the key role of PRC in the rapid modulation of metabolic functions in response to the status of the cell cycle.

## Introduction

Several essential cellular functions of mitochondria depend on a high degree of functional interaction between the nuclear and mitochondrial genomes. Of the hundred structural subunits that make up the oxidative phosphorylation (OXPHOS) complexes, 13 are encoded by the mitochondrial genome. The mechanisms governing the coordination of the multiple transcription factors involved in mitochondrial biogenesis have been partly explained by the discovery of the PGC-1 coactivator family [1]. Three members of this family – PGC1α, PGC1β and PRC – regulate several functions, including adaptative thermogenesis, glucidic metabolism, fatty acid oxidation and mitochondrial metabolism, *via* functional interactions with various transcriptional factors. Mitochondrial biogenesis is controlled by PGC mainly through interactions with nuclear respiratory factors, NRF-1 and NRF-2, and may be induced *via* p38 mitogen-activated protein kinase. In a cell-selective manner, the efficiency of the oxidative phosphorylation process may also be regulated by PGC through the transcriptional control of uncoupling proteins (UCPs) [2].

PGC-1α is expressed in organs and tissues with high energetic needs, such as heart, liver, skeletal muscle, brown adipose tissue, brain and kidney. In contrast, the expression of the PGC-1 related coactivator (PRC) depends on the cell cycle, and plays a role in the integration of pathways directing the mitochondrial respiratory function and cell growth [3]. PRC shares key structural motifs with PGC-1α, interacting with and transactivating the promoters of NRF-1 target genes in a similar manner [4], [5]. Other transcription factors targeted by PGC-1α have been studied as potential targets for PRC. Weak interactions between PRC, on one hand, and PPARγ, TRβ and RAR, on the other, reflect the divergence between PRC and PGC-1α; however, strong interactions between PRC and factors such as NRF-2, ERRα and CREB have recently been identified [6], [7]. The nuclear transcription factors involved in mitochondrial biogenesis are not exclusive to this process but contribute to integrating the expression of mitochondrial proteins with other cellular functions. Thus, PRC has been shown to control cell growth through mechanisms that seem to be independent of its effects on mitochondrial function [3]. PRC has also been described as a positive regulator of respiratory chain expression; however, the precise role played by PRC in the complex functions involved in mitochondrial biogenesis and cell growth remains to be elucidated.

Deregulation in the functional status of mitochondria can have a feedback effect on nuclear transcriptional machinery [8], [9]. It has been suggested that this retrograde signalling influences the bidirectional communication between the nucleus and mitochondria through a PGC-1 dependent pathway [10]. Mitochondrial functions are regulated by complex mechanisms in which nitric oxide (NO) is a key factor. The mitochondrial effect of NO is bi-phasic depending on its production level. Thus, NO is known to induce the production of reactive oxygen species (ROS) and trigger redox signalling [11]. It directly binds the haem-copper oxidases of complex IV and leads to reversible inhibition of the respiratory chain in an acute response. In a long term effect, a nitric oxide-cGMP-dependent pathway has been shown to control mitochondrial biogenesis through the PGC-1α pathway [12]. The overexpression of NO, cGMP, or eNOS (endothelial nitric oxide synthase) was found to dramatically increase the number of mitochondria in a range of cell lines. This overexpression, related to a high production of PGC-1α, leads to the formation of functionally efficient mitochondria in terms of oxidative phosphorylation. Interestingly, constitutive NO synthase – like eNOS – generates a low concentration of NO, which acts mainly as a second messenger to maintain cell homeostasis [13]. Moreover, eNOS was suspected of being one of the target genes of the retrograde signalling [14]. Since PRC presents the characteristics of an early gene product, it is likely to be involved in the rapid response to retrograde signals [7].

We have demonstrated in thyroid oncocytoma, which constitute a model of mitochondrial-rich tumours, a high rate of PRC induction, with no disruption of mitochondrial functions [15]. The PRC-induced effects were confirmed in the XTC.UC1 thyroid cell line, which thus offered a useful model for investigating the coordination of the nuclear and mitochondrial genomes [16], [17]. In these mitochondrial-rich cells, we found an increased expression of eNOS, clearly produced by follicular thyroid cells [18], [19]. To further appreciate the importance of the nuclear-mitochondrial crosstalk mediated by PRC, we searched for the cellular programs regulated by PRC in the oxidative XTC.UC1 human oncocytic thyroid cell line, using genome-wide expression profiles of PRC SiRNA *versus* those of negative control cells. We compared the pattern of PRC inhibition with the activation of mitogen-activated protein kinase (MAPK). We investigated the effect of NO on the induction of PRC in the oncocytic cells compared to a non-oxidative BCPAP cell line derived from a papillary thyroid carcinoma. We focussed on the various functional features of PRC compared to those of PGC-1α, especially with respect to the rapid response required to maintain mitochondrial and cell homeostasis.

## Results

### PRC-Mediated Mitochondrial Biogenesis Depends on the Level of Nitric Oxide Production

**Figure 1:**
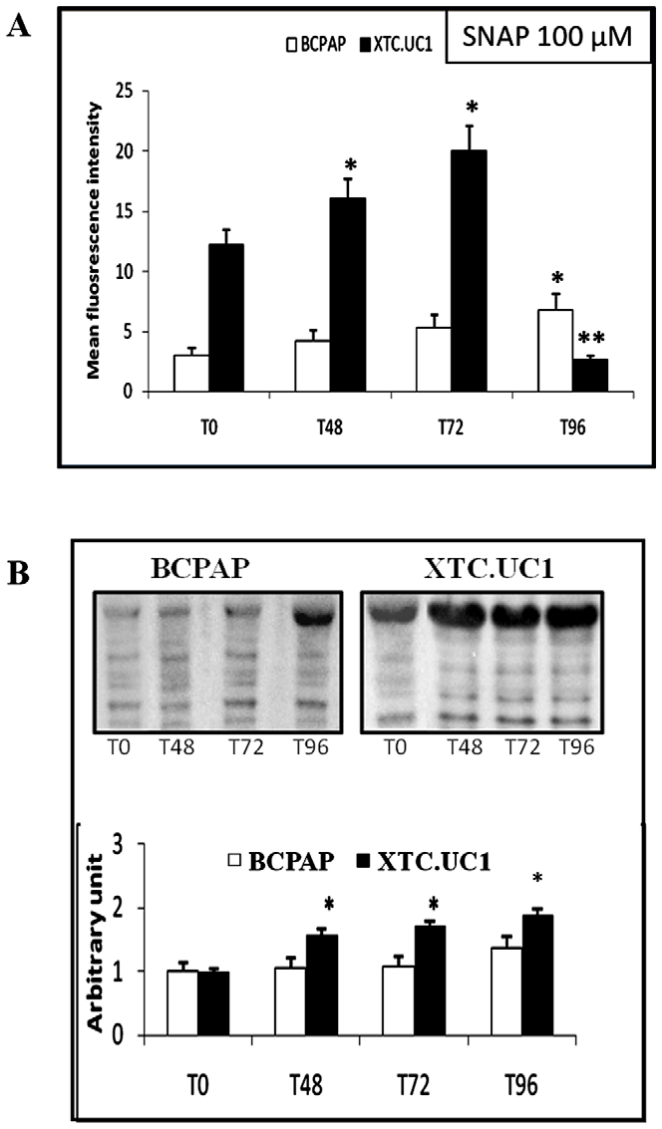
Effect of 100 µM SNAP treatment during 96 h on XTC.UC1 and BCPAP cell lines (*N* = 5 per cell line). 1A: Nitric oxide measurement with the FACScan cytometer (10 µM final DAF2/DA dye). Results are expressed in arbitrary fluorescent units as mean values±SEM. *P≤0.05 *versus* T0. **P≤0.05 *versus* T0 and T72. 1B: Evaluation of protein nitration by Western blotting using antibody raised against nitrotyrosine-modified proteins. Immunoblots are quantified by densitometric analysis and expressed in arbitrary units (relative to α-tubulin) as mean values±SEM. *P≤0.05 *versus* T0.

At T0, the endogenous NO was significantly higher (4.2 fold on average) in XTC.UC1 cells than in BCPAP cells (Figure 1A). Since identical results were obtained with the nitric oxide donor SNAP at concentrations of 50 µM and 100 µM, we considered only those of SNAP at 100 µM, this concentration being likely to produce 100 nM equivalent NO during several hours [20]. From T0 to T72, daily SNAP treatment induced comparable NO concentrations in the two cell lines, with a 1.6 fold increase in XTC.UC1 cells and a 1.7 fold increase in BCPAP cells. At T96, the use of the DAF-2/DA dye as an NO probe showed that the NO level decreased sharply for XTC.UC1 cells. This was associated with a 12-fold increase of ROS production, detected with a dihydroethidium probe, for XTC.UC1 cells at T96, whereas ROS production was stable at T48 and T72 (data not shown). The drastic decrease in the NO level measured by the DAF2 dye at T96 could be associated with the complete inhibition of complex IV activity and to the production of peroxynitrite. Dysfunctions in the respiratory chain could have induced an oxidative stress that, combined with the high NO level, may have produced peroxynitrite. However, contrary to probes measuring oxidative stress, the DAF2 probe was unable to detect peroxynitrite formation [21]. For BCPAP cells, NO production continuously increased at T96 up to 2.2 fold from T0. The profile of nitrotyrosine-modified proteins showed that the 100 µM SNAP treatment induced a significant increase in protein nitration in XTC.UC1 cells from T48 whereas protein nitration had not increased significantly in BCPAP cells at T96 (Figure 1B).

**Figure 2:**
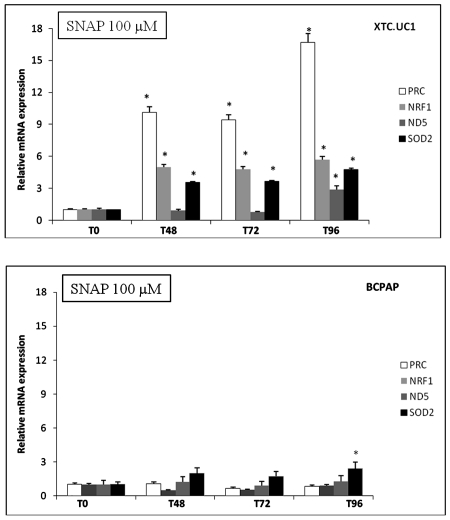
The expression of nuclear genes (PRC, NRF-1 and SOD2) and the mitochondrial gene (ND5) after 100 µM SNAP treatment of XTC.UC1 and BCPAP cells during 96 h. Data are expressed in relative units (mRNA copies number of a specific gene/copy number of β-globin mRNA) and expressed in terms of the T0 ratio as mean values±SEM. *P≤0.05 *versus* T0. *N* = 5 per cell line and time of treatment.

Regarding the mRNA expression of genes involved in mitochondrial biogenesis (NRF-1, ND5 and PRC) and mitochondrial ROS detoxification (SOD2), we showed that the 100 µM SNAP treatment induced nuclear gene expression from T48, whereas mitochondrial gene ND5 expression was significantly induced from T96 in XTC.UC1 cells (Figure 2). No significant induction of genes involved in mitochondrial biogenesis was observed in BCPAP cells up to T96 of SNAP treatment. However, the SOD2 expression level had increased significantly at T96 compared to the T0 level.

### PRC Expression Is Regulated at the Transcriptional Level

**Figure 3:**
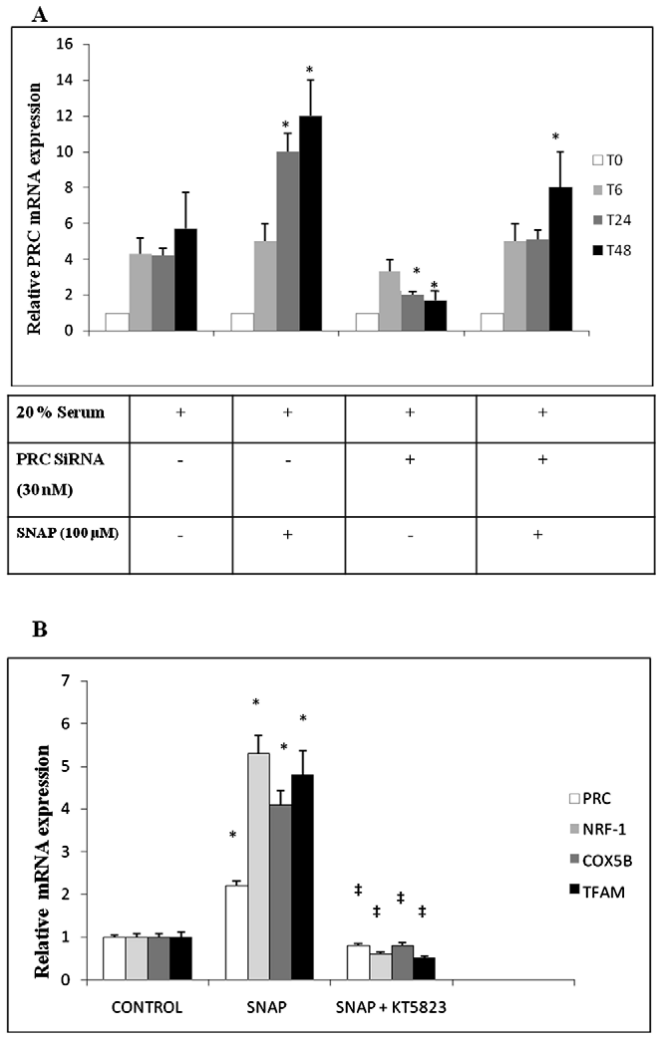
Effects of mRNA expression in XTC.UC1 cells during 20% serum induction and 100 µM SNAP and/or 30 nM PRC SiRNA treatment. Relative units (PRC mRNA copy number/β-globin copy number) expressed in terms of the T0 ratio as mean values±SEM. *N* = 5 per time of treatment. 3A: The variation of PRC mRNA during 48 h of the different treatments. Except for T48 SiRNA, all samples differed significantly from T0 (*P*≤0.05). *P≤0.05 *versus* T6. 3B: The variation of NRF-1, TFAM and COX5B mRNA at T24 of SNAP 100 µM and/or KT5823 1 µM (protein kinase G inhibitor) treatments compared to control T0. All samples differed significantly from T0 (*, *P*≤0.05) or from those with 100 µM SNAP treatment (‡, *P*≤0.05).

In XTC.UC1 cells synchronized by serum starvation for two days, we compared the PRC mRNA expression level during 48 h of 20% serum and PRC SiRNA and/or SNAP treatment (Figure 3A). The expression profile during 20% serum treatment was considered as a control. After 48 h of continuous SNAP treatment, there was a 2.2-fold increase of PRC induction compared to the 48 h control. SiRNA treatment reduced PRC expression by 70% at 48 h while SiRNA and SNAP used in combination led to PRC re-expression up to 1.4 times the 48 h control value. Moreover, PRC mRNA expression was rapidly re-induced to a significant level after 6 h of treatment with SNAP+SiRNA (5.6±0.6 relative PRC mRNA) compared to the level after 6 h of treatment with SiRNA alone (3.5±0.4 relative PRC mRNA, *P*≤0.05).

The expression of PRC and that of three genes involved in mitochondrial biogenesis (NRF-1, TFAM and COX5B) was significantly down-regulated when the NO/cGMP pathway was inhibited by the protein kinase G (PKG) inhibitor KT5823 at 1 µM (Figure 3B). This showed the involvement of the NO/cGMP pathway in the regulation of PRC expression, which decreased from 2.2±0.1 to 0.8±0.1 relative PRC mRNA with KT5823 treatment (*P*≤0.05).

### The Microarray Profile of Kinetic PRC SiRNA Reveals Nuclear-Mitochondrial Crosstalk

**Figure 4:**
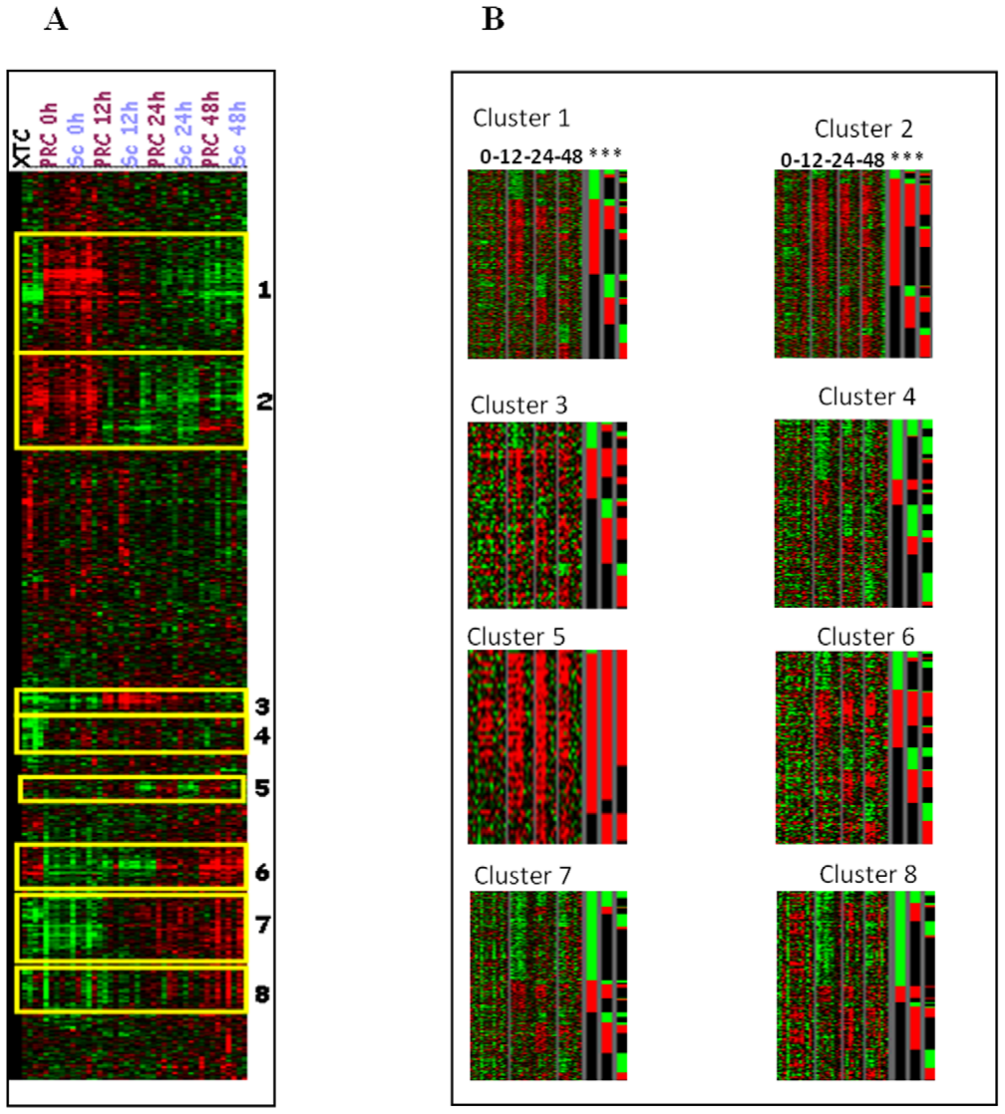
Microarray analysis of the XTC.UC1 cell line after transfection with either PRC SiRNA (PRC) or scramble as a negative control (Sc). The expression profiles were obtained at T12, T24 and T48 after SiRNA transfection and normalized to serum or scramble values (*N* = 4 per time). The XTC sample represents the gene expression profile of the cell line before transfection. 4A: Global clustering of PRC SiRNA and negative control (scramble) samples normalized by serum induction. Eight clusters were selected on significant differential genes for all time of SiRNA treatment (*P*≤0.03). 4B: Significant over- and under-expressed genes in SiRNA samples compared to negative control (scramble) samples for each of the 8 clusters previously identified by serum centring. At the right side of each cluster (***) are represented the genes differentially expressed (over-expressed in red, and under-expressed in green) at T12, T24 and T48 (*P*≤0.03) in PRC SiRNA compared to matched negative controls for time. Grey bars separate times of treatment as T0, T12, T24, and T48.

We examined the gene expression pattern of PRC SiRNA and control cells during the 48 h SiRNA treatment (Figure 4A). We had previously verified the level of PRC expression during the kinetics of inhibition by comparing PRC expression in SiRNA to a negative control by quantitative RT-PCR. PRC expression was inhibited from 42% (0 h and 12 h) to 70% at 24 h and 74% at 48 h. The absence of difference in the level of PRC inhibition between 0 h and 12 h may be explained by the dependence of PRC expression on cell cycle induction at 12 h even though PRC SiRNA had begun to act. Protein level measurements showed that SiRNA treatment was also able to inhibit PRC to more than 80% of the control after 24 h (Figure S1A).

Eight clusters of genes were differentially expressed between PRC SiRNA and the control during 48 h of 20% serum induction (Figure 4A). Gene ontology identified two clusters, 2 and 8, in which mitochondrial functions were enriched, and two clusters, 5 and 6, in which the cell cycle functions were enriched (Table 1). The comparison of the significant differential gene expression between PRC SiRNA and the negative control, in each cluster and at each time of treatment (Figure 4B), showed that some genes were negatively regulated by PRC at a given time and positively at another. This suggests that PRC regulates gene transcription through interactions with transcriptional factors in either a positive or a negative manner. We confirmed the differential expression of nine genes selected from clusters 2, 5 and 6 by quantitative RT-PCR analysis on 5 independent SiRNA experiments (Figure S1C). A Pearson correlation of 90% was found between results of microarray analysis and real-time RT–PCR.

**Table 1:** Description and gene ontology of the genes from the 8 clusters identified and normalized on serum induction, differentially expressed between PRC SiRNA and the negative control.

Cluster Number	Total genes	Differential gene expression PRC vs Negative control (p≤0.05)		Principal Functional annotations compared to 5225 filtered cDNA probes	P –value
		Over	Under		
1	786	209	127	G-protein activating pathway	0.0013
				Protein kinase and phosphatase activity	0.0017
				Regulation of signal transduction	0.0055
				Regulation of MAPK activity	0.0068
				Regulation of angiogenesis	0.0116
2	538	260	65	Oxidative phosphorylation	0.0001
				Response to stress	0.0001
				Carboxylic acid metabolism	0.0001
				Mitochondrial respiratory chain complex assembly	0.0011
				Fatty acid metabolic process	0.0039
				Nitrogen compound metabolic process	0.0049
				Mitochondrion organization and biogenesis	0.0245
3	131	38	23	Chromatin remodelling	0.0026
				Regulation of cytoskeleton organization	0.0026
				Cell to cell signalling	0.0121
				Cell junction assembly	0.0218
				Transcription coactivator activity	0.0193
4	243	21	114	Ribosome biogenesis and assembly	0.0001
				Vesicle organization and biogenesis	0.0001
				Protein-RNA complex assembly	0.0002
				Nucleic acid metabolic process	0.0006
				mRNA processing	0.0022
5	57	48	3	Protein targeting to membrane	0.0030
				DNA methyl transferase activity	0.0074
				Spindle elongation process	0.0107
				Cell cycle process	0.0302
				Regulation of S phase of mitotic cycle	0.0318
6	198	91	48	Regulation of M phase of cell mitotic cycle	0.0001
				Microtubule cytoskeleton organization	0.0001
				Chromosome segregation	0.0002
				Regulation of cyclin-dependent protein-kinase activity	0.0016
				Regulation of transcription factor activity	0.0041
7	386	61	123	Response to DNA damage	0.0001
				Response to endogenous stimulus	0.0001
				Base excision repair process	0.0006
				Mismatch repair process	0.0006
				Primary metabolism process	0.0013
8	267	35	69	Mitochondrial ribosome assembly	0.0001
				Regulation of RNA splicing	0.0003
				SnRNA binding	0.0062
				RNA modification	0.0062
				Folding	0.0120

**Figure 5:**
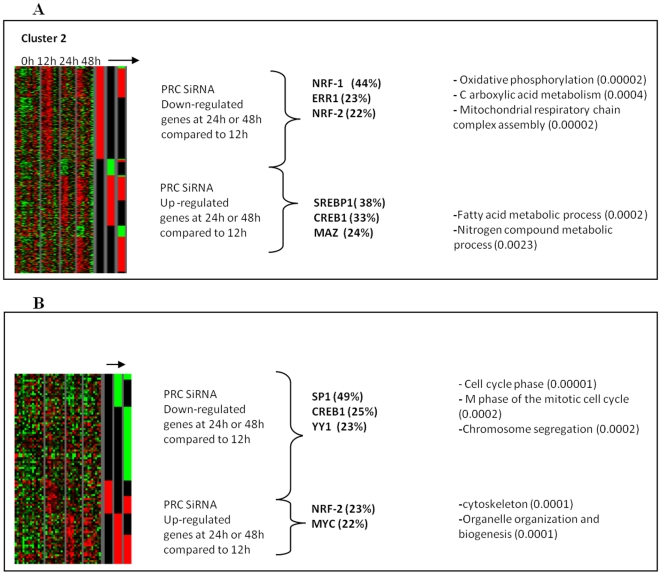
Focus on clusters 2 and 6 for PRC-regulated genes. 5A: PRC-sensitive under- and over-expressed genes in cluster 2. 5B: PRC-sensitive under- and over-expressed genes in cluster 6. The arrow represents the differential genes in SiRNA PRC compared to negative controls at times T12, T24 and T48. Using MSignDB software (GSEA, MIT, MI, USA), the promoters of selected genes, positively or negatively regulated by PRC, were explored for transcription factor motifs. The percentage of genes in which the motif for a selected transcription factor was found is indicated in brackets. Highly significant ontologies are shown (*P*≤0.01, GOMiner software [50]).

We searched for transcriptional factors involved in the PRC-regulating pathway in clusters 2 and 6 where ontological terms were enriched in mitochondrial metabolism and the cell mitotic cycle, respectively. Cluster 2 showed that PRC-positive regulation operated through NRF-1, NRF-2 and ERR1 interactions, whereas PRC-negative regulation acted mainly through SREBP1, CREB1 and MAZ interactions (Figure 5A). Cluster 6 showed that PRC-positive regulation depended on SP1, CREB1 and YY1 interactions whereas PRC-negative regulation depended on NRF-2 and MYC interactions (Figure 5B). The frequency of these motifs, ranging from 22–49% in the two gene clusters, was relevant of their involvement in the regulation of these genes compared to the relatively low frequency (2–15%) for the other genes of the microarray.

### Oxidative Phosphorylation Is Finely Regulated by PRC

**Figure 6:**
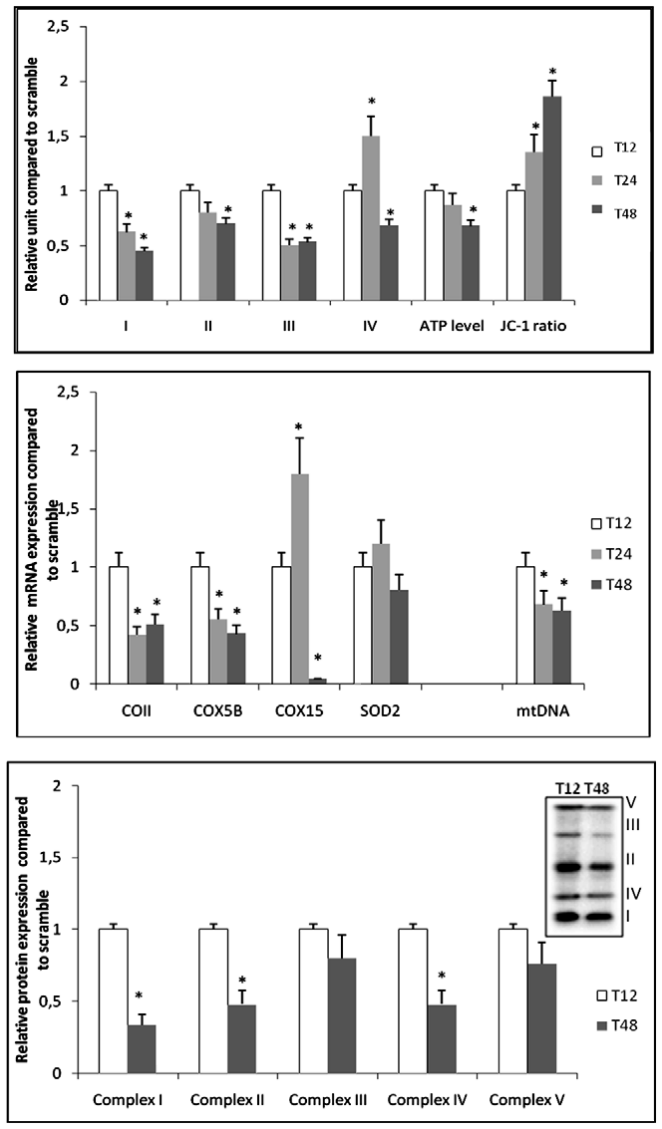
Functional and expression status of oxidative phosphorylation during PRC SiRNA treatment. T12 of serum induction during PRC SiRNA treatment was used as a reference to explore the efficient PRC inhibition at T24 and T48. (*N* = 5; * *P*≤0.05). 6A: Enzymatic activities of four complexes of the respiratory chain (I, II, III and IV), total cell ATP and JC-1 ratios were measured on PRC SiRNA treated cells and related to control scramble cells. The JC-1 ratio represents the coupling efficiency between the respiratory chain and ATP synthesis. 6B: cDNA and mtDNA copy numbers of selected genes during PRC inhibition. The mitochondrial gene (COII) and the nuclear genes (COX5B, COX15) correspond to subunits of complex IV of the respiratory chain. Their expression, associated to mitochondrial SOD2 gene expression and mtDNA copy number were measured at T12, T24 and T48 of PRC SiRNA treatment and referred to the control scramble. 6C: Western blot analysis of subunits from each of the five complexes of OXPHOS using the MS601 antibody cocktail (Mitosciences, Eugene, OR, USA) during SiRNA treatment against PRC. Complex I (NDUFB8, 20 KDa), complex II (Ip subunit, 30 KDa); complex III (Core 2:40 KDa); complex IV (COX2, 24 KDa); complex V (ATPsynthase F1α, 58 KDa), relative to α-tubulin (65 KDa). Measurements of SiRNA treatment at T12 and T48 are referred to the negative control.

Functional analysis of the respiratory chain complexes showed that complexes I and III activities were rapidly down-regulated when PRC inhibition was effective (T24 of SiRNA treatment referred to T12). This was associated with increased complex IV activity and better coupling between respiration and ATP synthesis (Figure 6A). At T48, the activity of each of the four complexes decreased; although the coupling was more efficient compared to that at T24 (JC-1 results), the ATP level was significantly lower at T48 than at T12. This may be related to a PRC-regulation of UCPs, as described for PGC-1α [2].

Regarding the expression of selected genes coding for complex IV subunits, the mitochondrial-encoded gene COII was down-regulated at T24, together with a decrease in mtDNA copy number (Figure 6B). The expression of nuclear-encoded complex IV subunit genes was more variable. There was a large variation in the expression of COX15 during PRC inhibition whereas the expression of COX5B decreased proportionally to that of COII. The expression of COX15 at T24 increased by 1.8 fold compared to that at T12, followed by a sharp decrease to 8% of the control value at T48. This may be related to the great difference in complex IV activity observed between T24 and T48. Taken together, these results showed that complexes I, III and IV were primarily regulated by PRC, in particular with regard to the expression of COX15, which may be associated with the increased activity of the complex IV in the first 12 h of PRC invalidation. Although these changes may have caused a functional default in the respiratory chain inducing oxidative stress, the expression of SOD2, a gene coding for a protein involved in superoxide detoxification, had not significantly decreased even at T48. However, the extension of the SiRNA treatment beyond T48 led to significantly down-regulated results (data not shown). The protein expression of several subunits from each OXPHOS complex decreased significantly at T48 for the three complexes I, II and IV, but the decrease was not significant for complexes III and V (Figure 6C). However, the gene and protein expressions of the different OXPHOS complexes were correlated so that global down-regulation was observed with PRC inhibition at T48 compared to T12.

### PRC Regulates Phosphatase/Kinase Activity

A phosphospecific antibody microarray for the MAPK signalling pathway was set up to compare the phosphorylation status of direct as well as indirect MAPK targets so as to determine whether PRC was required for the phosphorylation of MAPK targets. This antibody array included 93 highly specific and well-characterized phosphospecific antibodies for proteins in the MAPK pathway, each with six replicates (the raw data are shown in Table S1). The paired antibodies for the same, but unphosphorylated, target sites were also included in the array to allow determination of the relative level of phosphorylation. Using a cutoff ratio of 0.8, we identified 11 sites that were hypophosphorylated in PRC SiRNA cells at T48 compared with control cells. These sites were involved in the regulation of the activity of seven proteins: ASK1 (P83 and P966), TP53 (P18 and P46), ATF-2 (P69-51), c-JUN (P63) SAP/JNK (P183), ELK1 (P383 and P389), ESR1 (P167) and Stahmin-1 (P15). Except for Stahmin-1, all these proteins are involved in the redox-sensitive pathway controlled by the activation of Ask1 protein by phosphorylation. A significant decrease in the phosphorylation status of Ask1(Ser83) and Elk1(Ser383) was confirmed by western blot analysis on five independent PRC SiRNA treated cells compared to controls; the decrease was 32% for Ask1 and 58% for Elk1 (p<0.05) (Figure S1B).

## Discussion

PRC, a member of the family of PGC1 transcriptional coactivators, is expressed more abundantly in proliferative cells than in growth-arrested cells. Several studies have demonstrated the important role played by these coactivators in modulating tissue energetic resources [22]. In the mitochondrial-rich tumour model we investigated, PGC-1α expression remained very low, being induced neither by serum nor in compensation to PRC repression.

As reported by other authors [3], [6], we showed that the nuclear and mitochondrial genes of the respiratory chain were up-regulated in accordance with PRC induction in a time-dependent manner. We also observed an induction of SOD2 expression that had been previously shown to be regulated by PGC-1α [23]. However, the rapid action of the PKG inhibitor on both PRC expression and the nuclear genes controlling respiratory chain biosynthesis indicated that, in the XTC.UC1 cell model, mitochondrial biogenesis required the induction of the NO/PRC pathway. The increase in PRC expression observed with combined PRC invalidation and SNAP treatment may be explained by the role of PRC in the rapid response to change in the status of the cell cycle [7]. An activating process by phosphorylation, as described for PGC-1α, has not so far been reported for PRC. We observed that an activating process by nitration could rapidly modulate PRC expression. This mechanism has also been implicated in the regulation of several transcription factors, such as CREB, c-Myc, Jun or Fos [24], [25].

In XTC.UC1 cells, as well as in mitochondrial-rich tumours, we previously reported that the high cellular level of NO was related to the induction of eNOS synthesis in follicular thyroid cells [26]. The activation of eNOS by the PI3K-AKT pathway has been recently implicated in tumour maintenance [27]. This finding may be particularly relevant in the context of mitochondrial-rich tumours characterized by the absence of mutations usually described in thyroid tumours of follicular origin [28]. In contrast, BCPAP cells were unable to activate the PRC pathway, even when NO increased through SNAP treatment. Independently of the mitochondrial quantity and the specific growth media, there was a great difference in the mitochondrial expression profile and functions: while XTC.UC1 cells displayed an oxidative metabolism, BCPAP cells were mainly engaged in a glycolytic process [19]. This suggested that defects of the mitochondrial function in BCPAP cells may have induced a retrograde pathway modifying the status of cell phosphorylation [29]. Thus, the action of PRC in controlling the redox status should be taken into account in relation to cell transformation and cancer.

Our microarray data on PRC inhibition may be compared with the results of a recent study using a PRC ShRNA approach [3]. The use of SiRNA and ShRNA allowed us to distinguish between the different temporal roles played by PRC, with SiRNA revealing the short term effects and ShRNA indicating the chronic action of PRC inhibition. However in both studies, a 50% decrease of PRC mRNA was sufficient to demonstrate the specific regulatory functions of this coactivator with complete inhibition of its protein expression. The effects on the OXPHOS process were severe probably because of the lack of functional compensation by other members of this family of coactivators, as suspected following studies on PGC-1α and PGC-1β null mice [30], [31]. Contrary to reports on ShRNA PRC knockdown [3], we identified a decrease in mtDNA content that may be primarily related to the decrease in OXPHOS activity. We postulate that long term PRC inhibition by ShRNA could induce compensatory mechanisms to maintain mitochondrial functions used for other cellular metabolisms. Using SiRNA, we have defined the positive as well as the negative regulations mediated by PRC through specific transcription factors. These may represent the direct and indirect transcriptional effects of the PRC coactivator. Thus, the PRC factor should be seen as a physiological integrator of energetic metabolism in other biological processes.

Focusing on clusters ontology, we showed that negative PRC regulation could be exerted on the biogenesis of the cytoskeleton and organelles. This should be compared with the surprising regulation of mitochondrial complex IV activity during SiRNA treatment. While complex IV activity has been previously related to PRC expression [3], the time-related regulation we report has never been described so far. Indeed, the analysis of the expression of some mitochondrial and nuclear-encoded genes of complex IV showed that COX15 expression varied in a manner opposite to that of other genes. This differential expression identified on microarray data was confirmed by real-time quantitative PRC analysis. The role of COX15 in the assembly of human complex IV has recently been emphasized [32]. COX15 was also found to be an essential component of the catalytic centre of the complex. This PRC regulation seems to be independent of a direct NO effect on the haem-copper of complex IV as there is a correlation between the activity of this complex and the expression of COX15 mRNA. We postulate that PRC ensures a rapid adaptation to the cellular environment through the regulation of a few target genes such as COX15. It separately regulates the expression of OXPHOS subunits, leading to a rapid modulation of mitochondrial functionality by integrating the regulation of complex IV activity with coupling efficiency and ROS detoxification processes.

We associated PRC functions with some PGC-1α regulated functions, such as chromatin remodelling, RNA splicing, translation and angiogenesis [33], [34]. Regulation of the cell cycle by PRC could be compared to the regulation of PGC-1α and PGC-1β by nutritional and hormonal signals as well as by circadian pacemakers [35]. It has recently been described that the ubiquitous PRC member could support the basal energetic cellular needs when cycling progression from the G1 phase [3], [6], [36]. The rapid regulation of complexes I and III, as well as the specific modulation of complex IV activity we observed, confirmed the key role of PRC for the rapid adaptation of cellular energy demand. The effects of PRC on the M-phase of the cell cycle have not been described so far. We suggest that PRC coordinates cell-cycle phases with mitochondrial metabolism through both positive and negative interactions with CREB1 and NRF-2 transcription factors. Previous studies have clearly associated CREB1 and NRF2 with the regulation of genes involved in energetic metabolism as well as the cell cycle [37], [38]. Even though these results are supported by the frequency of representation for motifs associated with these transcription factors in specific clusters, they need to be confirmed by results obtained with the chromatine-immunoprecipitation technique.

Some pathways, such as those of the phosphatase/kinase activities and vesicle organization, also seem to be specifically related to PRC [3]. Interestingly, the MAPK profile of PRC-inhibited cells showed a significant decline in the phosphorylation level of proteins involved in the ASK1 signalling pathway. ASK1, a serine/threonine protein kinase, is activated by the exposure of cells to various stimuli, including tumour necrosis factor-α, Fas ligand and hydrogen peroxide [39]. It lies upstream of a major redox-sensitive pathway leading not only to the induction of apoptosis but also to cell proliferation and differentiation [40]. The majority of proteins involved in the control of the cell cycle have been shown to be redox-sensitive with functions in the G1 and G2/M phases [41]. Since the G2/M phase is in a more reduced state than the G1 phase, oxidant-sensitive proteins may be temporally regulated by the oscillation of the intracellular redox environment [42]. We postulate that PRC may represent the redox sensor enabling the initiation of the retrograde signalling pathway, especially in a cellular model presenting a balance between the production of nitric oxide and superoxide. Interestingly, eNOS activity has also been shown to be redox-sensitive [43]. Further studies will be needed to clarify the temporal role of PRC in the integrative regulation of cell metabolism.

In conclusion, we have identified several new pathways regulated by the PRC coactivator. Focusing on OXPHOS, we showed that the PRC-regulation of this process differed from that of PGC-1α. The nuclear OXPHOS genes were tightly controlled by PGC-1α but less so by PRC. Nevertheless, other genes such as COX15 were found to be more specifically regulated by PRC than by PGC-1α. This precision control of mitochondrial energy metabolism should be placed in the context of the complex regulation of the cell cycle. Our study showed that the eNOS/PRC signalling pathway can modulate the cell cycle by regulating the intracellular redox status. The action of PRC, complementary to that of other PGC-1 factors, should therefore be further explored, especially in the case of metabolic diseases.

## Materials and Methods

### Cell Lines and SNAP Treatment

Two human thyroid cell lines, XTC.UC1 and B-CPAP, were used. The XTC.UC1 cell line was established from an oncocytic cell thyroid carcinoma. The growth medium consisted of Dulbecco's modified Eagle medium (DMEM) supplemented with 10% foetal calf serum (Seromed, Biochrom AG, Berlin, Germany), 100 U/mL penicillin, 100 mg/mL streptomycin, 0.25 mg/mL fungizone, and 10 mU/mL thyrotropin (TSH) (Sigma-Aldrich, Saint Louis, MO, USA). The B-CPAP cell line was established from a human papillary thyroid carcinoma cultured in RPMI-1640 medium with 10% foetal calf serum, 100 U/mL penicillin, 100 mg/mL streptomycin, 0.25 mg/mL fungizone. Except for the TSH and the foetal calf serum, all the products were obtained from Gibco BRL (Life Technologies, Paisley, United Kingdom).

The two cell lines were treated once a day for four days with the nitric oxide donor SNAP (S-nitroso-N-acetyl-D,L-penicillamine, EMD, San Diego, CA, USA) at a final concentration of either 50 or 100 µM in the selected medium. Except for the microarray analysis, which was performed in quadruplicate (*N* = 4), all the assays were performed in quintuplicate (*N* = 5). To control the mediating effect of NO/cGMP on PRC expression, cells were pre-treated with 1 µM of the protein kinase G inhibitor KT5823 (EMD, San Diego, CA, USA) 30 minutes before SNAP treatment (100 µM) during 24 h.

### Flow Cytometry Analysis

XTC.UC1 cells were exposed at 37°C for 20 minutes to 5 µg/mL JC-1 (5,5′,6,6′-tetrachloro-1,1′,3,3′-tetraethylbenzimidazolcarbocyanine) or for 30 minutes at pH 7 to 10 µM DAF-2/DA (4,5-diaminofluorescein diacetate) before trypsin treatment. The two dyes were purchased from EMD, San Diego, CA, USA.

The cells were then harvested, washed twice with PBS, and analyzed on a FACScan flow cytometer (Becton Dickinson, Franklin Lakes, NJ, USA). Cultured cells, untreated with dyes, were used as a negative control to determine the FACS gating. JC-1 emits red fluorescence (aggregates) when sequestered in the mitochondrial membrane of healthy cells and emits green fluorescence (monomers) when released into the cytoplasmic compartment of the cell. At the depolarized membrane potential (−100 mV), the JC-1 green monomer emission peaks at about 527 nm. At the hyperpolarized membrane potential (−140 mV) the red emission of the JC-1 aggregates shifts towards 590 nm. The red/green ratio represents the modification of mitochondrial functionality during treatment. DAF-2/DA is a cell-permeable derivative of DAF-2 presenting an emission peak at about 515 nm, and which reacts neither with stable oxidized forms of NO, such as NO_2_^−^ and NO_3_^−^, nor with other reactive oxygen species, such as O_2_^−^, H_2_O_2_, and ONOO^−^ [44].

### Quantitative PCR Analysis

MtDNA and cDNA quantifications were performed on a Chromo4 apparatus (Bio-Rad, Hercules, CA, USA) using SYBR Green I dye as a fluorescent signal (iQ sybrGreen supermix, Bio-Rad), according to the manufacturer's recommendations. For mtDNA quantification, primers were located at the following nucleotide positions: forward, 3254–3277 (in the ND1 gene), and reverse, 3412–3391 (in the tRNA^Leu^ gene). The mtDNA copy number was expressed in terms of the copy number of a 110-bp fragment of the β-globin gene. For cDNA quantification, the expression of 16 selected genes (14 nuclear genes: PRC, PGC-1α, NRF-1, COX5B, COX15, TFAM, SOD2, ALDH6A1, OSGIN2, PRKAA2, CDCA3, CDC14B, CDC25C and β-GLOBIN and two mitochondrial genes, COII and ND5) was measured by real-time quantitative RT-PCR using the protocol previously described [45] and the primers listed in Table 2.

**Table 2:** Primer sequences used for real-time quantitative RT-PCR.

ALDH6A1	Forward	**5′- CCA-GTC-CCA-CCT-GGT-ATT-CA-3′**
	Reverse	**5′- CCC-GAC-CAA-TGA-CCT-CAT-TG-3′**
CDCA3	Forward	**5′- GTG-GAG-AGC-TCT-CCA-CAG-CC-3′**
	Reverse	**5′- CCC-TTG-AGG-GCT-TGT-CGG-AG-3′**
CDC14B	Forward	**5′- CCG-CTG-AAC-TTG-GCA-ATG-GG-3′**
	Reverse	**5′- GCT-GCA-ATT-TGC-TCT-TTT-TCC-G-3′**
CDC25C	Forward	**5′- GTC-ACC-TGG-ATT-CTT-CAG-GA-3′**
	Reverse	**5′- GCA-GAT-GAA-CTA-CAC-ATT-GC-3′**
COII	Forward	**5′ - AAC-AAA-CGA-CCT-AAA-ACC-TG – 3′**
	Reverse	**5′ - GTG-AAC-TAC-GAC-TGC-TAG-AA – 3′**
COX5B	Forward	**5′ - CCA-AAG-GCA-GCT-TCA-GGC-AC – 3′**
	Reverse	**5′ - CAA-GGA-AGA-CCC-TAA-TCT-AG – 3′**
COX15	Forward	**5′ - CCT-CTC-GAT-GGT-AGA-TTG-GC – 3′**
	Reverse	**5′ - CGG-AGG-GAG-TGC-AGT-GAC-AG – 3′**
ND5	Forward	**5′ - GGG-GAT-TGT-GCG-GTG-TGT-G – 3′**
	Reverse	**5′ - CTT-CTC-CTA-TTT-ATG-GGG-GT – 3′**
NRF-1	Forward	**5′ - GGA-GTG-ATG-TCC-GCA-CAG-AA – 3′**
	Reverse	**5′ - CGC-TGT-TAA-GCG-CCA-TAG-TG -3′**
OSGIN2	Forward	**5′- GAA-ATG-GAC-CCT-CAG-GAA-TA-3′**
	Reverse	**5′- GGA-CCT-TTA-CCA-AGA-ACT-AC-3′**
PGC-1	Forward	**5′ - ACT-CAA-GTG-GTG-CAG-TGA-CC – 3′**
	Reverse	**5′ - CTG-GGT-ACT-GAG-ACC-ACT-GC – 3′**
PRC	Forward	**5′- GAT-CAG-AGC-AGC-GCT-GGG - 3′**
	Reverse	**5′- CAC-TAG-CAG-CTC-TCT-CCC-C – 3′**
PRKAA2	Forward	**5′- GGA-GAA-CAT-CAA-TTA-ACA-GG-3′**
	Reverse	**5′- CTG-CTG-AAA-GAG-CCG-CCT-GG-3′**
SOD2	Forward	**5′ - GCT-GCA-CCA-CAG-CAA-GCA-CC – 3′**
	Reverse	**5′ - CCA-GCA-ACT-CCC-CTT-TGG-GT – 3′**
ΤFΑΜ	Forward	**5′ –CCG-AGG-TGG-TTT-TCA-TCT-GT- 3′**
	Reverse	**5′ –CAG-GAA-GTT-CCC-TCC-AAC-GC-3′**
β-GLOBIN	Forward	**5′ - GGT-GAA-CGT-GGA-TGA-AGT-TG – 3′**
	Reverse	**5′ - GAG-CCA-GGC-CAT-CAC-TAA-AG – 3′**

### Respiratory Chain Complex Activities and Cellular ATP

Respiratory chain complex activities were measured in cell lysates, thermostatically maintained at 37°C, using a Beckman spectrophotometer (DU800, Beckman Coulter, Fullerton, CA, USA). Cells were resuspended in a cell buffer containing 250 mM saccharose, 20 mM Tris, 2 mM EDTA, 1 mg/ml BSA, pH 7.2 (50 µl/10^6^ cells).

To measure the activity of respiratory complexes I and II, the cells were first disrupted by freezing in liquid nitrogen, followed by rapid thawing at 37°C. Complex I activity was immediately assayed on cell lysate (0.5×10^6^ cells) in a KH_2_PO_4_ buffer (100 mM, pH 7.4), containing 1 mM KCN, 2 mM NaN_3_, and 0.1 mM ubiquinone-1. After incubation for 5 min, the reaction was started by adding 0.3 mM NADH and the rate of disappearance of NADH was monitored at 340 nm. Rotenone (5 µM) was then added to determine the background rate. Complex II (succinate ubiquinone reductase) activity was measured as described elsewhere [46], except that the background rate was measured by the addition of thenoyltrifluoroacetone (200 µM). Complex III (after a freeze-thaw cycle), complex IV and citrate synthase (CS) activities were assayed as described previously [47].

Twenty µl of CellTiter-Glo reagent (Promega, Madison, CA, USA) were added to 3.10^5^ cells, growing in 200 µl of culture medium, to measure the cellular ATP level. An ATP curve was prepared as recommended by the manufacturers. Plates were agitated for 2 min and incubated for 10 min at room temperature before measuring the luminescence (Lumat 9507, Berthold Technologies, Bad Wildbad, Germany). The ATP level was expressed as ng per mg of total cell protein measured by the BCA method (Pierce, Rockford, IL, USA).

### PRC SiRNA

To knock down PRC expression in the XTC.UC1 cells, three predesigned PRC SiRNAs (from Applied Biosystems, Foster city, CA, USA) were tested in comparison to a random negative control SiRNA (scramble, #4635). The PRC SiRNA (#121729) was chosen for at least 70% of PRC mRNA expression knockdown. For the study, 30 nM of this PRC siRNA were transfected in the XTC.UC1 cells using siPORT NeoFX (Applied Biosystems) as recommended by the manufacturer. To synchronize the cell cycle for SNAP and/or PRC SiRNA treatments, the cells were serum-starved for two days before adding SiRNA during a 48 h period. After 6 h, 24 h and 48 h serum induction, XTC.UC1 cells were harvested for RNA or phospho-protein isolation. To synchronize the cell cycle for genome-wide expression analysis, the cells were serum-starved during 7 h before replacing the medium with 20% SVF medium for 48 h. After 12 h, 24 h and 48 h serum induction corresponding to 19 h, 31 h and 55 h SiRNA treatment, XTC.UC1 cells were harvested for protein, DNA and RNA isolation. We refer to the times of 20% serum induction (T0, T12, T24 and T48) instead of the times of SiRNA treatment (T7, T19, T31 and T55) in the figures and the text.

The PRC expression level, analyzed by quantitative RT-PCR, normalized by β-globin expression, was considered relevant when PRC mRNA inhibition reached 70% of that of the negative control (scramble) with a significant effect on COX5B, the PRC-regulated gene.

### Microarray Analysis

cDNA microarray slides were prepared at the Transcriptome Core Facility of the University of Nantes (INSERM U915, France), using a set of 20,000 oligonucleotides with full functional characterization and content referencing (Ocimum Biosolutions, Hyderabad, India). RNA amplification, cDNA labelling and hybridization from XTC.UC1 cells were performed using the protocols recommended by the Transcriptome Core Facility (http://cardioserve.nantes.inserm.fr/ptf-puce/). Slides were analyzed with Axon GenePix 4.0 software (Axon, Union City, CA, USA) after scanning on a ScanArray Express II scanner (Packard Bioscience, Billerica, MA, USA). Data are available in the GEO database (GSE 14282) and are confirmed to be MIAME compliant for raw and normalized data as well as for protocols used. The hierarchical clustering of the genes was computed on median-gene-centred and log-transformed data using average linkage and uncentred correlation distances. Computations and visualization were performed using Cluster and TreeView programs [48]. EASE and Gene Set analyses were used to determine the statistical likelihood that *a priori* families of genes were over-represented and differentially expressed [49]. Differentially expressed genes were defined as those well detected in both groups and with absolute PLS (partial least-squares) loadings >3.0 and t-test values of *P*<0.0002 (FDR = 0.001). In addition, genes with an absolute value of mean expression differences <40% were excluded from further analyses. Gene ontology enrichments in gene lists were searched for using GOMiner [50]. The most abundant gene ontology terms, representing at least 5% of the genes in the lists, with P-values lower than 0.05, were considered for interpretation.

The over-representation of transcription-factor binding sites (TFBS) in promoter sequences was investigated with MSignDB software (GSEA, MIT, MI, USA). We added two more position-weight matrices to this collection: for the NRF1 transcriptional factor, we aligned 9 sequences of known NRF-1 binding sites [51]; for the oestrogen-related receptor alpha (ERR1), the position-weight matrix used was that described by Sladek *et al.* [52]. The percentage of representation of a given transcription factor was compared to its representation in the rest of the microarray.

### Western Blot Analysis

Cells were mixed with a buffer containing 10 mmol/L HEPES (pH 7.9), 1.5 mmol/L MgCl_2_, 10 mmol/L KCl, 0.5% NP-40 and a protease inhibitor cocktail, and then centrifuged at 13,000 g for 5 min. Supernatants were collected and the protein concentration was determined by the BCA method (Pierce, Rockford, IL, USA). Samples containing 30 µg total protein were applied to 12% SDS-PAGE gel electrophoresis and hybridized with a cocktail of five antibodies against human nuclear-encoded subunits of the OXPHOS process: Complex I (NDUFB8, 20 KDa), complex II (Ip subunit, 30 KDa); complex III (Core2, 40 KDa); complex IV (COX2, 24 KDa); complex V (ATPsynthase F1α, 58 KDa), at a dilution of 1/500 (MS 601, MitoSciences, Eugene, OR, USA). The level of nitration-modified protein was measured using a high-activity rabbit polyclonal anti-nitrotyrosine antibody at a dilution of 1/250 (US Biological, Swampscott, MA, USA). The PRC protein level was measured using a rabbit polyclonal antibody at the dilution of 1/5000. This antibody was produced against a human peptide (1520–1534) we had previously selected (Eurogentec, Seraing, Belgium). The antibody revealed a unique 170 kDa band on immunoblots. Ask1 and Elk1 phosphorylation status was evaluated using at the dilution of 1/500, anti- Ask1(Phospho-Ser83) and Elk1(Phospho-Ser383) antibodies and anti- unphosphorylated Ask1 and Elk1 antibodies (all from Abcam, Cambridge, UK).

After overnight incubation with primary antibodies, membranes were washed before incubation with the corresponding horseradish peroxidase (HRP)-conjugated secondary antibody, which was detected with a chemiluminescent detection system (ECL Plus, Amersham Biosciences, Fairfield, CT, USA).

### Phospho-Specific Protein Microarray Analysis

The phospho-specific protein microarray was obtained from Full Moon Biosystems (Sunnyvale, CA, USA). Protein microarray analysis was carried out using the protocol provided by the manufacturer. Briefly, 100 µg of cell lysate in 50 µl of reaction mixture were labelled with 1.43 µl biotin in 10 µg/µl N,N-dimethyformamide. The resulting biotin-labelled proteins were diluted 1∶20 in a coupling solution before being applied to the array for conjugation. To prepare the antibody microarray, it was first blocked with a blocking solution for 30 min at room temperature, rinsed with Milli-Q grade water for three minutes, and then dried by centrifugation. Finally, the array was incubated with the biotin-labelled cell lysates at room temperature during four hours. After the array slide was washed thrice with 50 ml of 1x wash solution for 10 min each, the conjugated-labelled protein was detected using Cy3-streptavidin.

### Data Analysis

Except for the microarray analyses, data are represented as mean values±SEM, with *N* representing the number of experiments. Statistical analyses were performed by a one-way analysis of variance, the Mann-Whitney *U* test, and Tukey's HSD test. Differences were considered to be statistically significant at *P*≤0.05. The Pearson chi-squared test was used to compare quantitative RT-PCR to microarray results.

## Supporting Information

Figure S1 Western blot analysis and differential gene expression data during SiRNA treatment against PRC in XTC.UC1 cell line (N = 5, *P≤0.05). S1A: Western blot analysis of PRC expression at T24 and T48 of SiRNA treatment compared to control (β-Tubulin). PRC antibody is raised to the (1520–1534) subregion of the molecule and reveals a unique band near to 170 kDa on immunoblots. S1B: Analysis of phosphorylation status for Ask1(Phospho-Ser83) and Elk1(Phospho-Ser383) after 48 h of PRC SiRNA treatment compared to control. For each protein, two antibodies are used raised to phosphorylated and unphosphorylated subregions revealing 155kDa (Ask1) and 46 kDa (Elk1) bands on immunoblots. S1C: Comparison of differential gene expression data obtained by microarrays and real-time RT-PCR after 48 h PRC SiRNA treatment. The upper and lower limits of each box stand for the upper and the lower quartiles, respectively; bold lines represent medians; whiskers represent extreme measurements. Regulation of genes from cluster 2 (TFAM, COX5B, COX15), cluster 5 (LDH6A1, OSGIN2, PPKAA2) and cluster 6 (CDCA3, CDC14B, CDC25C) were confirmed on 5 independent SiRNA experiments.
(5.52 MB TIF)

Table S1 Phospho-specific Antibody Array analysis of PRC SiRNA XTC.UC1 cells and control. Cell lysates were labeled with Biotin and incubated with antibody array, separately. Arrays were washed and labeled proteins were detected by Cy3-streptavidin. Signal represents the average signal intensity of six replicates. CV: coefficient of variation.
(0.23 MB DOC)
