# Supplementary figures and images for: PGC-1-Related Coactivator Modulates Mitochondrial-Nuclear Crosstalk through Endogenous Nitric Oxide in a Cellular Model of Oncocytic Thyroid Tumours
Source: PLoS One. 2009 Nov 23;4(11):e7964. doi: 10.1371/journal.pone.0007964 (PMC2776512; doi:10.1371/journal.pone.0007964)

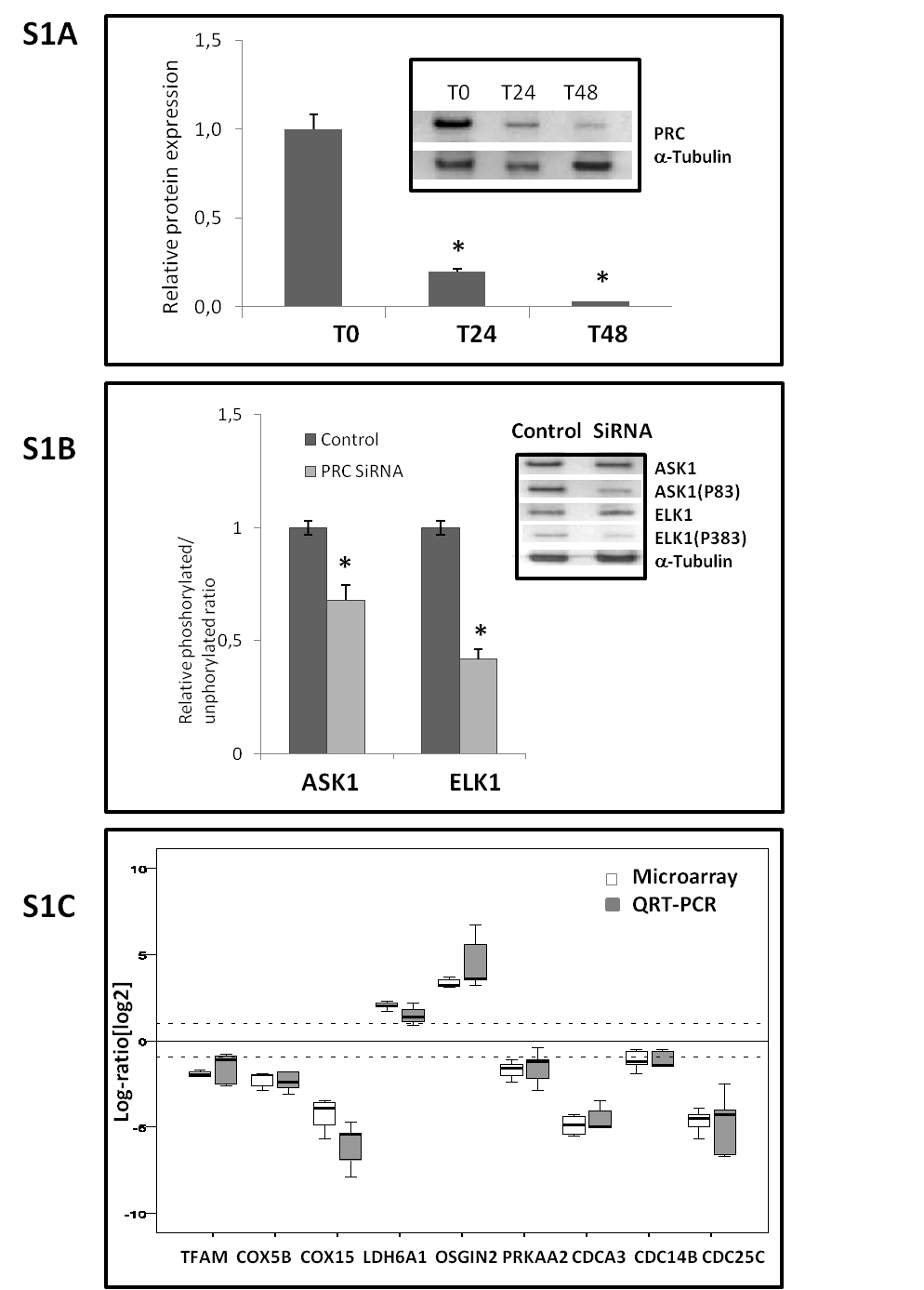

Supplement: Figure S1 — Western blot analysis and differential gene expression data during SiRNA treatment against PRC in XTC.UC1 cell line (N = 5, *P≤0.05). S1A: Western blot analysis of PRC expression at T24 and T48 of SiRNA treatment compared to control (β-Tubulin). PRC antibody is raised to the (1520–1534) subregion of the molecule and reveals a unique band near to 170 kDa on immunoblots. S1B: Analysis of phosphorylation status for Ask1(Phospho-Ser83) and Elk1(Phospho-Ser383) after 48 h of PRC SiRNA treatment compared to control. For each protein, two antibodies are used raised to phosphorylated and unphosphorylated subregions revealing 155kDa (Ask1) and 46 kDa (Elk1) bands on immunoblots. S1C: Comparison of differential gene expression data obtained by microarrays and real-time RT-PCR after 48 h PRC SiRNA treatment. The upper and lower limits of each box stand for the upper and the lower quartiles, respectively; bold lines represent medians; whiskers represent extreme measurements. Regulation of genes from cluster 2 (TFAM, COX5B, COX15), cluster 5 (LDH6A1, OSGIN2, PPKAA2) and cluster 6 (CDCA3, CDC14B, CDC25C) were confirmed on 5 independent SiRNA experiments. (5.52 MB TIF) [file pone.0007964.s001.tif]
