# Supplementary material for: PGC-1-Related Coactivator Modulates Mitochondrial-Nuclear Crosstalk through Endogenous Nitric Oxide in a Cellular Model of Oncocytic Thyroid Tumours
Source: PLoS One. 2009 Nov 23;4(11):e7964. doi: 10.1371/journal.pone.0007964 (PMC2776512; doi:10.1371/journal.pone.0007964)
Supplement: Table S1 — Phospho-specific Antibody Array analysis of PRC SiRNA XTC.UC1 cells and control. Cell lysates were labeled with Biotin and incubated with antibody array, separately. Arrays were washed and labeled proteins were detected by Cy3-streptavidin. Signal represents the average signal intensity of six replicates. CV: coefficient of variation. (0.23 MB DOC) [file pone.0007964.s002.doc]

|  | **control** | | **PRC SiRNA** | |
| --- | --- | --- | --- | --- |
| **Name** | **Signal** | **CV** | **Signal** | **CV** |
| 4E-BP1(Ab-36) | 588 | 0.07 | 1155 | 0.04 |
| 4E-BP1(Ab-45) | 860 | 0.04 | 1206 | 0.04 |
| 4E-BP1(Phospho-Thr36) | 1501 | 0.05 | 1326 | 0.05 |
| 4E-BP1(Phospho-Thr45) | 740 | 0.04 | 875 | 0.04 |
| ASK1(Ab-83) | 1270 | 0.05 | 1275 | 0.05 |
| ASK1(Ab-966) | 951 | 0.13 | 970 | 0.06 |
| ASK1(Phospho-Ser83) | 2784 | 0.04 | 1986 | 0.04 |
| ASK1(Phospho-Ser966) | 2912 | 0.03 | 2183 | 0.06 |
| ATF2(Ab-112 or 94) | 731 | 0.05 | 1415 | 0.05 |
| ATF2(Ab-62 or 44) | 667 | 0.07 | 1180 | 0.07 |
| ATF2(Ab-69 or 51) | 651 | 0.04 | 1088 | 0.05 |
| ATF2(Ab-71 or 53) | 902 | 0.06 | 1029 | 0.06 |
| ATF2(Ab-73 or 55) | 765 | 0.03 | 1228 | 0.03 |
| ATF2(Phospho-Ser112 or 94) | 1746 | 0.09 | 1515 | 0.09 |
| ATF2(Phospho-Ser62 or 44) | 1549 | 0.05 | 1473 | 0.05 |
| ATF2(Phospho-Thr69 or 51) | 2489 | 0.04 | 1588 | 0.04 |
| ATF2(Phospho-Thr71 or 53) | 917 | 0.04 | 1401 | 0.04 |
| ATF2(Phospho-Thr73 or 55) | 916 | 0.05 | 1316 | 0.05 |
| ATF4(Ab-245) | 1210 | 0.05 | 1520 | 0.05 |
| ATF4(Phospho-Ser245) | 1121 | 0.03 | 1284 | 0.03 |
| c-Jun (Phospho-Thr239) | 737 | 0.05 | 1356 | 0.05 |
| c-Jun(Ab-170) | 1138 | 0.07 | 1481 | 0.07 |
| c-Jun(Ab-239) | 995 | 0.03 | 1441 | 0.03 |
| c-Jun(Ab-243) | 1404 | 0.05 | 1372 | 0.05 |
| c-Jun(Ab-63) | 707 | 0.04 | 1430 | 0.04 |
| c-Jun(Ab-73) | 2564 | 0.03 | 2436 | 0.03 |
| c-Jun(Ab-91) | 671 | 0.05 | 1331 | 0.05 |
| c-Jun(Ab-93) | 1144 | 0.04 | 1592 | 0.04 |
| c-Jun(Phospho-Ser243) | 1773 | 0.02 | 1463 | 0.02 |
| c-Jun(Phospho-Ser63) | 1965 | 0.04 | 1544 | 0.04 |
| c-Jun(Phospho-Ser73) | 1295 | 0.03 | 1430 | 0.03 |
| c-Jun(Phospho-Thr91) | 844 | 0.03 | 1414 | 0.03 |
| c-Jun(Phospho-Thr93) | 2318 | 0.02 | 1400 | 0.02 |
| c-Jun(Phospho-Tyr170) | 1916 | 0.01 | 1446 | 0.01 |
| c-Kit(Ab-721) | 958 | 0.08 | 1076 | 0.08 |
| c-Kit(Phospho-Tyr721) | 1829 | 0.06 | 1656 | 0.06 |
| CREB(Ab-129) | 957 | 0.06 | 1143 | 0.06 |
| CREB(Ab-133) | 532 | 0.04 | 1357 | 0.05 |
| CREB(Phospho-Ser129) | 1477 | 0.05 | 1545 | 0.05 |
| CREB(Phospho-Ser133) | 1364 | 0.05 | 1321 | 0.05 |
| eIF2 alpha (Phospho-Ser51) | 1214 | 0.05 | 1429 | 0.05 |
| eIF2 alpha(Ab-51) | 791 | 0.07 | 937 | 0.01 |
| eIF2 alpha(Phospho-Ser51) | 1137 | 0.02 | 1313 | 0.02 |
| eIF2((Phospho-Ser51) | 1266 | 0.05 | 1317 | 0.05 |
| elF4E(Ab-209) | 939 | 0.03 | 1169 | 0.03 |
| elF4E(Phospho-Ser209) | 1191 | 0.02 | 1435 | 0.02 |
| Elk-1(Ab-383) | 877 | 0.02 | 1268 | 0.02 |
| Elk1(Ab-389) | 2061 | 0.03 | 1749 | 0.03 |
| Elk1(Ab-417) | 1134 | 0.01 | 1368 | 0.01 |
| Elk-1(Phospho-Ser383) | 2571 | 0.02 | 1503 | 0.02 |
| Elk1(Phospho-Ser389) | 2382 | 0.04 | 1605 | 0.04 |
| Elk1(Phospho-Thr417) | 1658 | 0.08 | 1474 | 0.08 |
| Estrogen Receptor-alpha (Ab-104) | 1722 | 0.02 | 1175 | 0.02 |
| Estrogen Receptor-alpha (Ab-106) | 4893 | 0.06 | 3429 | 0.06 |
| Estrogen Receptor-alpha (Ab-118) | 574 | 0.04 | 1229 | 0.04 |
| Estrogen Receptor-alpha (Ab-167) | 1086 | 0.04 | 1258 | 0.04 |
| Estrogen Receptor-alpha (Phospho-Ser104) | 1841 | 0.04 | 1342 | 0.04 |
| Estrogen Receptor-alpha (Phospho-Ser106) | 1513 | 0.02 | 1283 | 0.02 |
| Estrogen Receptor-alpha (Phospho-Ser118) | 1750 | 0.09 | 1353 | 0.09 |
| Estrogen Receptor-alpha (Phospho-Ser167) | 2253 | 0.06 | 1562 | 0.06 |
| FAK(Ab-397) | 975 | 0.02 | 1223 | 0.02 |
| FAK(Ab-861) | 1080 | 0.05 | 1132 | 0.05 |
| FAK(Ab-925) | 601 | 0.03 | 1102 | 0.03 |
| FAK(Phospho-Tyr861) | 1825 | 0.05 | 1487 | 0.05 |
| FAK(Phospho-Tyr925) | 1146 | 0.03 | 1221 | 0.03 |
| GAPDH | 581 | 0.02 | 1013 | 0.02 |
| Histone H2A.X(Ab-139) | 2386 | 0.02 | 2491 | 0.02 |
| Histone H2A.X(Phospho-Ser139) | 1740 | 0.03 | 1165 | 0.03 |
| Histone H3.1(Ab-10) | 1187 | 0.05 | 1146 | 0.05 |
| Histone H3.1(Phospho-Ser10) | 1930 | 0.04 | 1523 | 0.04 |
| HSF1(Ab-303) | 474 | 0.03 | 1084 | 0.03 |
| HSF1(Phospho-Ser303) | 1787 | 0.02 | 1310 | 0.02 |
| HSP27(Ab-15) | 638 | 0.05 | 1078 | 0.05 |
| HSP27(Ab-78) | 561 | 0.06 | 985 | 0.06 |
| HSP27(Ab-82) | 1382 | 0.06 | 1127 | 0.06 |
| HSP27(Phospho-Ser15) | 1681 | 0.03 | 1507 | 0.03 |
| HSP27(Phospho-Ser78) | 1926 | 0.01 | 1738 | 0.01 |
| HSP27(Phospho-Ser82) | 1885 | 0.04 | 1619 | 0.04 |
| IRS-1(Ab-307) | 613 | 0.03 | 1050 | 0.03 |
| IRS-1(Ab-312) | 443 | 0.06 | 1231 | 0.06 |
| IRS-1(Ab-636) | 595 | 0.05 | 1141 | 0.05 |
| IRS-1(Ab-639) | 4454 | 0.08 | 2387 | 0.08 |
| IRS-1(Phospho-Ser307) | 870 | 0.04 | 1120 | 0.04 |
| IRS-1(Phospho-Ser312) | 1576 | 0.02 | 1359 | 0.02 |
| IRS-1(Phospho-Ser636) | 2296 | 0.04 | 1590 | 0.04 |
| IRS-1(Phospho-Ser639) | 1480 | 0.01 | 1278 | 0.01 |
| JunB(Ab-259) | 1556 | 0.04 | 1223 | 0.04 |
| JunB(Ab-79) | 1003 | 0.06 | 1292 | 0.06 |
| JunB(Phospho-Ser259) | 2463 | 0.01 | 1456 | 0.01 |
| JunB(Phospho-Ser79) | 1937 | 0.07 | 1413 | 0.07 |
| JunD(Ab-255) | 745 | 0.01 | 1125 | 0.01 |
| JunD(Phospho-Ser255) | 2403 | 0.04 | 1505 | 0.04 |
| MEF2A(Ab-312) | 1758 | 0.05 | 1405 | 0.05 |
| MEF2A(Ab-319) | 1354 | 0.06 | 1617 | 0.06 |
| MEF2A(Phospho-Thr312) | 1255 | 0.02 | 1029 | 0.02 |
| MEF2A(Phospho-Thr319) | 1152 | 0.25 | 1316 | 0.25 |
| MEK1(Ab-217) | 917 | 0.05 | 1217 | 0.05 |
| MEK1(Ab-221) | 642 | 0.03 | 1205 | 0.03 |
| MEK1(Ab-291) | 568 | 0.02 | 1076 | 0.02 |
| MEK1(Phospho-Ser217) | 1033 | 0.02 | 1244 | 0.02 |
| MEK1(Phospho-Ser221) | 1447 | 0.04 | 1432 | 0.04 |
| MEK1(Phospho-Thr291) | 912 | 0.02 | 1342 | 0.02 |
| MEK-2(Ab-394) | 1907 | 0.03 | 1772 | 0.03 |
| MEK-2(Phospho-Thr394) | 2377 | 0.04 | 1154 | 0.04 |
| Met(Ab-1234) | 1705 | 0.02 | 1355 | 0.02 |
| Met(Ab-1349) | 1403 | 0.02 | 1198 | 0.02 |
| Met(Phospho-Tyr1234) | 2207 | 0.03 | 1452 | 0.03 |
| Met(Phospho-Tyr1349) | 404 | 0.05 | 891 | 0.05 |
| MKK3(Ab-189) | 779 | 0.02 | 1059 | 0.02 |
| MKK3(Phospho-Ser189) | 1694 | 0.01 | 1191 | 0.01 |
| Myc(Ab-358) | 1402 | 0.02 | 1646 | 0.02 |
| Myc(Ab-373) | 1043 | 0.02 | 1591 | 0.02 |
| Myc(Ab-58) | 1348 | 0.03 | 1509 | 0.03 |
| Myc(Phospho-Ser373) | 994 | 0.02 | 1294 | 0.02 |
| Myc(Phospho-Thr358) | 1415 | 0.09 | 1348 | 0.03 |
| Myc(Phospho-Thr58) | 2002 | 0.03 | 1547 | 0.02 |
| P38 MAPK(Ab-182) | 880 | 0.10 | 1023 | 0.03 |
| P38 MAPK(Phospho-Thr180) | 1140 | 0.03 | 1309 | 0.03 |
| P38 MAPK(Phospho-Tyr182) | 1489 | 0.04 | 1332 | 0.04 |
| p44/42 MAP Kinase(Ab-202) | 882 | 0.03 | 1211 | 0.03 |
| p44/42 MAP Kinase(Ab-204) | 677 | 0.02 | 1110 | 0.02 |
| p44/42 MAP Kinase(Phospho-Thr202) | 1320 | 0.03 | 1497 | 0.03 |
| p44/42 MAP Kinase(Phospho-Tyr204) | 2019 | 0.02 | 1734 | 0.02 |
| p53(Ab-15) | 721 | 0.01 | 816 | 0.01 |
| p53(Ab-18) | 595 | 0.04 | 804 | 0.04 |
| p53(Ab-315) | 1129 | 0.02 | 1172 | 0.02 |
| p53(Ab-33) | 1290 | 0.02 | 1184 | 0.02 |
| p53(Ab-37) | 955 | 0.04 | 1149 | 0.04 |
| p53(Ab-46) | 865 | 0.02 | 1004 | 0.02 |
| p53(Ab-6) | 689 | 0.06 | 1083 | 0.06 |
| p53(Ab-9) | 825 | 0.02 | 955 | 0.02 |
| p53(Phospho-Ser15) | 1526 | 0.05 | 1378 | 0.05 |
| p53(Phospho-Ser315) | 1722 | 0.04 | 1481 | 0.04 |
| p53(Phospho-Ser33) | 1556 | 0.04 | 1507 | 0.04 |
| p53(Phospho-Ser37) | 1050 | 0.01 | 1308 | 0.01 |
| p53(Phospho-Ser46) | 2416 | 0.03 | 1707 | 0.03 |
| p53(Phospho-Ser6) | 1734 | 0.02 | 1540 | 0.02 |
| p53(Phospho-Ser9) | 1922 | 0.05 | 1548 | 0.05 |
| p53(Phospho-Thr18) | 2317 | 0.06 | 1754 | 0.06 |
| PKC delta (Phospho-Ser645) | 1585 | 0.06 | 1544 | 0.06 |
| PKC delta(Ab-645) | 730 | 0.03 | 1087 | 0.03 |
| PKC theta (Phospho-Ser676) | 533 | 0.01 | 984 | 0.01 |
| PKC theta(Ab-676) | 614 | 0.04 | 1031 | 0.04 |
| PKC-delta (Phospho-Ser645) | 1728 | 0.03 | 1562 | 0.03 |
| PKC-theta(Phospho-Ser676) | 509 | 0.06 | 994 | 0.06 |
| Pyk2(Ab-402) | 923 | 0.05 | 1184 | 0.05 |
| Pyk2(Phospho-Tyr402) | 1093 | 0.08 | 1113 | 0.08 |
| Raf1(Ab-259) | 1632 | 0.04 | 1624 | 0.04 |
| Raf1(Ab-338) | 2216 | 0.02 | 1582 | 0.02 |
| Raf1(Phospho-Ser259) | 2346 | 0.04 | 1536 | 0.04 |
| Raf1(Phospho-Ser338) | 1830 | 0.01 | 1653 | 0.01 |
| SAPK/JNK(Ab-183) | 1236 | 0.04 | 1540 | 0.04 |
| SAPK/JNK(Ab-185) | 982 | 0.06 | 1005 | 0.06 |
| SAPK/JNK(Phospho-Thr183) | 1530 | 0.04 | 1494 | 0.04 |
| SAPK/JNK(Phospho-Tyr185) | 1625 | 0.07 | 1464 | 0.07 |
| SEK1/MKK4(Ab-261) | 901 | 0.01 | 1053 | 0.01 |
| SEK1/MKK4(Phospho-Ser80) | 1387 | 0.04 | 1418 | 0.04 |
| SEK1/MKK4(Phospho-Thr261) | 2043 | 0.02 | 1496 | 0.02 |
| Src(Ab-418) | 1080 | 0.06 | 1167 | 0.05 |
| Src(Ab-529) | 1724 | 0.02 | 1391 | 0.02 |
| Src(Phospho-Tyr418) | 1856 | 0.25 | 1403 | 0.25 |
| Src(Phospho-Tyr529) | 1370 | 0.05 | 1354 | 0.05 |
| Stathmin 1(Ab-15) | 959 | 0.03 | 1099 | 0.03 |
| Stathmin 1(Ab-24) | 592 | 0.02 | 1050 | 0.02 |
| Stathmin 1(Ab-37) | 624 | 0.02 | 1255 | 0.02 |
| Stathmin 1(Phospho-Ser15) | 2943 | 0.04 | 1647 | 0.04 |
| Stathmin 1(Phospho-Ser24) | 1315 | 0.02 | 1351 | 0.02 |
| Stathmin 1(Phospho-Ser37) | 1631 | 0.03 | 1325 | 0.03 |
| Tau(Ab-181) | 590 | 0.04 | 993 | 0.04 |
| Tau(Ab-205) | 1854 | 0.02 | 1135 | 0.02 |
| Tau(Ab-212) | 900 | 0.02 | 1179 | 0.02 |
| Tau(Ab-214) | 1090 | 0.03 | 1336 | 0.03 |
| Tau(Ab-231) | 1500 | 0.05 | 1317 | 0.03 |
| Tau(Ab-235) | 1637 | 0.02 | 1345 | 0.02 |
| Tau(Ab-262) | 916 | 0.01 | 1200 | 0.04 |
| Tau(Ab-356) | 756 | 0.02 | 1053 | 0.04 |
| Tau(Ab-396) | 903 | 0.02 | 919 | 0.02 |
| Tau(Ab-404) | 1627 | 0.03 | 1273 | 0.05 |
| Tau(Ab-422) | 1476 | 0.02 | 1337 | 0.02 |
| Tau(Phospho-Ser214) | 1839 | 0.09 | 1249 | 0.09 |
| Tau(Phospho-Ser235) | 1508 | 0.03 | 1055 | 0.03 |
| Tau(Phospho-Ser262) | 1731 | 0.10 | 1288 | 0.10 |
| Tau(Phospho-Ser356) | 1339 | 0.03 | 1410 | 0.03 |
| Tau(Phospho-Ser396) | 1149 | 0.04 | 1041 | 0.04 |
| Tau(Phospho-Ser404) | 1218 | 0.03 | 1178 | 0.03 |
| Tau(Phospho-Ser422) | 824 | 0.02 | 1167 | 0.02 |
| Tau(Phospho-Thr181) | 823 | 0.03 | 821 | 0.03 |
| Tau(Phospho-Thr205) | 939 | 0.02 | 929 | 0.02 |
| Tau(Phospho-Thr212) | 1627 | 0.01 | 1479 | 0.05 |
| Tau(Phospho-Thr231) | 1234 | 0.04 | 1152 | 0.07 |
| Negative conrol | 334 | 0.01 | 452 | 0.02 |
